# Soluble fibrin inhibits monocyte adherence and cytotoxicity against tumor cells: implications for cancer metastasis

**DOI:** 10.1186/1477-9560-4-12

**Published:** 2006-08-22

**Authors:** John P Biggerstaff, Brandy Weidow, Jacqueline Vidosh, Judith Dexheimer, Shonak Patel, Pretesh Patel

**Affiliations:** 1 Biological Imaging Unit, University of Tennessee, 10515 Research Drive, # 300, Knoxville, TN 37932, USA

## Abstract

**Background:**

Soluble fibrin (sFn) is a marker for disseminated intravascular coagulation and may have prognostic significance, especially in metastasis. However, a role for sFn in the etiology of metastatic cancer growth has not been extensively studied. We have reported that sFn cross-linked platelet binding to tumor cells via the major platelet fibrin receptor αIIbβ3, and tumor cell CD54 (ICAM-1), which is the receptor for two of the leukocyte β2 integrins (α_L_β2 and a_M_β2). We hypothesized that sFn may also affect leukocyte adherence, recognition, and killing of tumor cells. Furthermore, in a rat experimental metastasis model sFn pre-treatment of tumor cells enhanced metastasis by over 60% compared to untreated cells. Other studies have shown that fibrin(ogen) binds to the monocyte integrin α_M_β2. This study therefore sought to investigate the effect of sFn on β2 integrin mediated monocyte adherence and killing of tumor cells.

**Methods:**

The role of sFn in monocyte adherence and cytotoxicity against tumor cells was initially studied using static microplate adherence and cytotoxicity assays, and under physiologically relevant flow conditions in a microscope perfusion incubator system. Blocking studies were performed using monoclonal antibodies specific for β2 integrins and CD54, and specific peptides which inhibit sFn binding to these receptors.

**Results:**

Enhancement of monocyte/tumor cell adherence was observed when only one cell type was bound to sFn, but profound inhibition was observed when sFn was bound to both monocytes and tumor cells. This effect was also reflected in the pattern of monocyte cytotoxicity. Studies using monoclonal blocking antibodies and specific blocking peptides (which did not affect normal coagulation) showed that the predominant mechanism of fibrin inhibition is via its binding to α_M_β2 on monocytes, and to CD54 on both leukocytes and tumor cells.

**Conclusion:**

sFn inhibits monocyte adherence and cytotoxicity of tumor cells by blocking α_L_β2 and α_M_β2 binding to tumor cell CD54. These results demonstrate that sFn is immunosuppressive and may be directly involved in the etiology of metastasis. Use of specific peptides also inhibited this effect without affecting coagulation, suggesting their possible use as novel therapeutic agents in cancer metastasis.

## Background

A relationship between cancer and abnormalities of the coagulation system has been recognized for over 100 years [1]. Thromboembolic disease (usually of unknown etiology), refractory to anticoagulant therapy, may be an early detectable sign of an underlying cancer, which could precede the onset of observable cancer by months or years. Although many cancer patients exhibit clinically significant hemostatic abnormalities, about 50% of all patients (>90% with metastases) have abnormal laboratory coagulation parameters [1], including soluble fibrin (sFn) [2-4], which may also be an early marker undiagnosed malignancy [5]. The presence of sFn in blood has, until recently, been considered a benign marker for the presence of an ongoing coagulopathy. However, we have reported a direct role for sFn in melanoma metastasis in an experimental model [6]. Furthermore, recent reports from *in vivo *studies suggest that pulmonary metastasis is reduced in fibrinogen deficient animals [7]. Several studies suggest that sFn may be a prognostic marker in cancer [8,9], but no clinical studies have been performed to directly associate sFn with increased metastasis. However, there a number of reports describing a direct clinical association with other coagulation proteins, including tissue factor (TF: reviewed in [10]), Factor VIII [11], and thrombin (reviewed in [12]).

A role for coagulation in tumor biology is further inferred by the anti-tumor effects of anticoagulant drugs, such as heparin, warfarinin[13] and other coumarin derivatives [14], thrombin inhibitors [15,16], and in a number of both experimental and spontaneous animal tumor models [17-20]. However, these therapies also increase the risk of bleeding due to inhibition of normal clotting.

**Figure 1:**
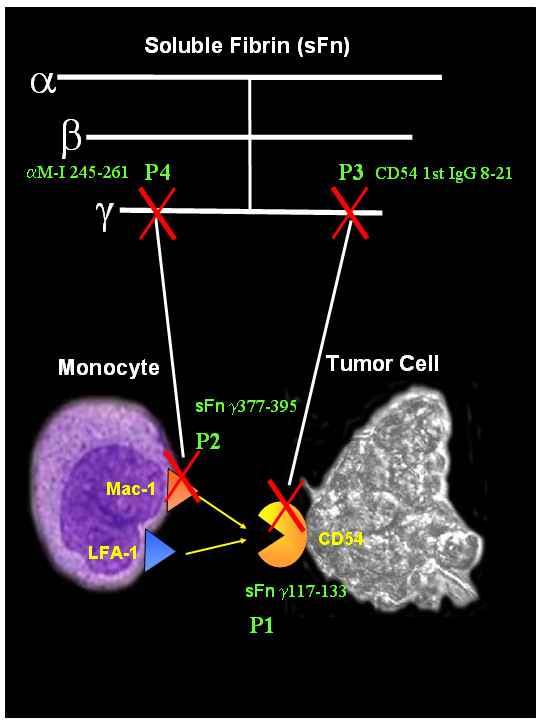
Schematic diagram showing the amino acid sequences, sites of origin and effector molecules for four peptides (designated P1 – P4) reported to inhibit fibrin(ogen) binding to α_M_β2 (orange) and CD54 (blue).

Fibrin(ogen) binds to a wide range of cellular receptors, including two of the leukocyte β2 integrins, α_M_β2 (MAC-1) and α_X_β2 (p150,95) [21], and the β2 integrin receptor, CD54 (ICAM-1), which are important mediators of monocyte diapedesis. Several peptides corresponding to potential fibrin(ogen) – α_M_β2 binding domains have been identified, but the most inhibitory one reported, designated P2C (^377^YKMKKTTMKIIPFNRLTIG^395^)[22] is considered to be a major fibrin(ogen) γ-chain binding site for α_M_β2 and α_X_β2. The major fibrin(ogen) γ-chain binding site reported on α_M_β2 is in the α_M _I-domain (^245^KFGDPLGYEDVIPEADR^261^)[23]. Binding of fibrin(ogen) to α_M_β2 correlates with differentiation and is involved in cellular signaling producing rapid perturbations in cytosolic Ca^2+ ^resulting in upregulation of α_M_β2 [24]. The major fibrin(ogen) binding site on CD54 is in the 1^st ^Immunoglobulin domain (^8^KVILPRGGSVLVTC^21^)[25], which binds to the fibrinogen γ-chain (^117^NQKIVNLKEKVAQLEA^133^)[21]. Figure 1 is a schematic diagram showing the sequences and specificities of each of these peptides on the sFn γ-chain and on α_M_β 1 and CD54.

In order for fibrin(ogen) to bind to cells it must first undergo a conformational change to expose these sites which may occur when fibrinogen is immobilized on endothelial cells, resulting in enhanced monocyte adherence [26]. This would augment the immune response to inflammatory sites, since plasma fibrinogen is not adherent to cells. However, in these studies, consideration was not given to the elevated plasma levels of sFn (which is likely to be conformationally altered) in the blood of many patients with cancer and other conditions.

The primary hypothesis in this study was, therefore, that sFn bound to monocytes and to tumor cells would result in inhibition, rather than augmentation of cellular adherence, and consequently cellular cytotoxicity. We further hypothesized that blockade of sFn binding to α_M_β2 and CD54 using the above peptides would result in restoration of these effector functions without affecting normal clotting.

## Methods

### Venepunture

Thirty milliliters of peripheral blood were drawn from normal, healthy volunteers into 3.2% sodium citrate vacutainers (Becton Dickinson VACUTAINER™ Systems, Rutherford, New Jersey). Whole blood diluted by adding 20 μl of blood into 180 μl 1% crystal violet stain in 0.5% acetic acid. A leukocyte count was performed in an improved Neubauer counting chamber.

Venepuncture and cell isolation for this study was performed in accordance with the University of Tennessee Institutional Review Board approval (IRB# 6788B).

### Preparation of Soluble Fibrin (sFn)

sFn is made in the required amount for each experiment. To produce sFn monomer, an excess of GPRP-NH2 is added (4 mM final). To make 1 ml of sFn solution, 50 μl fibrinogen (10 mg/ml; plasminogen-, fibronectin-, and factor XIII free; American Diagnostica Inc., Greenwich, Connecticut), 84 μl of the fibrin polymerization inhibitor Gly-Pro-Arg-Pro-amide (24 mM; GPRP-NH2; Sigma Chemical Company), followed by 1.25 μl thrombin (100 U/ml; Sigma Chemical Company) were added to 865 μl of RPMI 1640.

For experiments investigating peptide inhibition of sFn binding to monocytes and tumor cells, FITC-labeled fibrinogen (Molecular Probes, Eugene, OR) was used in place of unlabeled fibrinogen above.

### Culture of tumor cells

The A375 human amelanotic malignant melanoma cell line was maintained in continuous cell culture. Cells were detached from plastic using trypsin/EDTA (0.25%: Hyclone, Logan, Utah) and washed in RPMI 1640 tissue culture medium containing 10% fetal bovine serum (10% FBS) centrifuged at 200 × g, resuspended in 5 ml of10% FBS, and counted. For static adherence assays, 4 × 10^4 ^cells were added to the wells of a 96 well flat-bottomed microtiter plate and incubated for 24–48 or until confluent. For cytotoxicity experiments, cells were incubated with 5 μl of Calcein-AM (Molecular Probes [27]) for 30 min, washed, counted and used at appropriate concentrations in a 96 well round-bottomed plate, to which effector cells were added. For flow experiments, the cell suspension was added to 40 mm glass coverslips together with 10% FBS in petri dishes and incubated until nearly confluent (24–48 h).

### Isolation of mononuclear cells

Six tubes of citrated blood were diluted 1:1 with RPMI, and 15 ml layered over 8 ml Lymphoprep™ in plastic universal containers. These discontinuous density gradients were centrifuged for 25 min at 450 × *g *and the mononuclear cell interface removed using a sterile plastic pasteur pipette. The cells were washed three times by resuspension and recentrifugation at 200 × *g *for 10 min in RPMI/FBS so as to remove platelets. The final cell pellets were resuspended in 10 ml RPMI. A cell count was performed by adding 20 μl of blood into 180 μl 0.5% trypan blue vital stain, and cells were counted in an improved Neubauer counting chamber.

### Purification of monocytes

Mononuclear cells obtained from the Lymphoprep™ density gradient were adjusted to 2 × 10^6 ^cells/ml, and aliquots of 5 × 10^7 ^cells added to 75 cm^2 ^serum coated tissue culture flasks. The flasks were incubated at 37°C, in 5% CO_2_, for 1.5 h in order to allow monocyte adherence to the plastic. The non-adherent cells (lymphocytes) were decanted. A cell count was performed on the final cell suspension an aliquot were dried onto microscope slides for analysis of cell purity. The flasks containing the remaining adherent cells (monocytes) were rinsed twice with RPMI and 10 ml of 3 mmol/L EDTA in RPMI/FBS added. The flasks were then incubated at 37°C for 12 min, or until the cells have detached from the plastic (determined by observation under an inverted microscope). The cells were removed from the flasks using a 5 ml pipette, transferred into universal containers, and washed three times in 10% FBS to remove any residual EDTA. Cell purity was determined by differential counting of lymphocytes and monocytes using May-Grunwald/Giemsa stain (Sigma). It was important that the monocytes were used in experiments immediately to avoid re-adherence to the plastic, which would result in a decreased cell yield. In 19 experiments, the mean monocyte purity was 92 ± 5%.

### Preparation of soluble fibrin solution

To produce 1 mL of sFn monomer solution, 50 μl fibrinogen (10 mg/ml; plasminogen-, fibronectin-, and factor XIII free; American Diagnostica Inc., Greenwich, Connecticut), 84 μl of the fibrin polymerization inhibitor Gly-Pro-Arg-Pro-amide (24 mM; GPRP-NH2; Sigma Chemical Company), followed by 1.25 μl thrombin (100 U/ml; Sigma Chemical Company) was added to 865 μl of RPMI 1640.

### Cellular adherence assay

Tumor cells were detached from plastic using trypsin (0.25%)/EDTA (0.1 μM) and washed in cell culture medium. Forty thousand cells (in 200 μl 10% FBS) were added to each well of a 96 well, flat bottomed tissue culture plate and incubated at 37°C until confluent. Six wells were trypsinized and the cells mixed 1:1 with Trypan blue stain (0.5% in PBS), and counted in a hemocytometer. The mean count was recorded. Adherent cells were left untreated (controls) or incubated with sFn, fibrinogen (0.5 mg/ml), GPRP-NH2 (4 mM) or thrombin (0.125 U/ml).

After washing with RPMI, 5 μl of Calcein AM (Molecular Probes, Eugene, OR) stock solution in DMSO were added to 5 ml of effector cell preparation (Leukocytes; 2 × 10^6 ^cells/ml) and incubated for 30 min at 37°C. Effector cells were left untreated (controls) or incubated with sFn or fibrinogen (0.5 mg/ml), GPRP-NH2 (4 mM) or thrombin (0.125 U/ml). Total fluorescence was determined by addition of 200 μl of fluorescently labeled effector cells to three wells and fluorescence measured. Minimal fluorescence (blank) was measured in three wells containing adherent cells to which only RPMI is added. After a further wash, cells were made to 1 × 10^6^/ml and 200 μl added to appropriate wells, and incubated at 37°C for 1 h. The plates were washed three times with RPMI followed by addition of 200 μl of 0.5 M NaOH to lyse the cells. The supernatants were removed into black 96-well microtiter plates and fluorescence was measured on a Perkin-Elmer Victor 3 plate reader. Specific adherence was determined by:

(Test - Blank/Total - blank)*100%.

### Antibody blocking of cellular adherence

Lyophilized murine anti-human monoclonal blocking antibodies directed against α_L_β2 (clone 25.3) and CD54 (clone 84H10) were obtained from Beckman-Coulter (Miami, FL), as were purified immunoglobulin matched isotypic controls (IgG1 (mouse) Clone 679.1Mc7). Blocking monoclonal anti-α_M_β2 (clone ICRF44) was obtained from PharMingen (BD Biosciences, San Jose, CA). All antibody inhibition experiments were performed using pre-incubation of target cells with 5 μg antibody/10^6 ^cells. Experiments were performed in triplicate wells and the results were expressed as the mean ± SD for three separate experiments).

### Measurement of cellular cytotoxicity

Tumor cells were detached from plastic using trypsin (0.25%)/EDTA (0.1 μM), washed and resuspended in 2 ml of culture medium and counted. Cell aliquots were untreated or pretreated as described in the previous section, depending on the experiment to be performed. The cell concentration was adjusted to 1 × 10^5 ^cells/ml and 100 μl added to the wells of a round-bottomed 96 well microtiter plate. One hundred microliters of untreated or appropriately pre-incubated monocytes were added to the tumor cells in appropriate wells at an effector target cell ratios of 20:1, and the plates were centrifuged at 250 × g for 10 min and 100 μl of supernatant carefully removed into the appropriate wells of an optically clear 96 well flat bottomed plate. LDH activity was measured using a standard kit. (Roche Molecular Biochemicals, Indianapolis, Indiana). One hundred microliters of reaction mixture were added to the wells and incubated at room temperature for 30 min in the dark. The absorbance of each well was measured using a Perkin-Elmer Victor 3 plate reader equipped to measure absorbance. Specific cytotoxicity was determined by (test release- blank/total release – blank)*100%. Initial experiments were performed to determine the optimum time course and effector:target (E:T) cell ratio for monocyte cytotoxicity (data not shown). Based on these results, sFn inhibition studies were performed using and E:T ratio of 20:1 in an 18 h assay, which was consistent with other reports [28,29].

### Monocyte adherence to tumor cells under flow conditions

To simulate the fluid shear stresses present *in vivo*, coverslips containing Calcein AM labeled, adherent A375 were loaded into the FCS2 stage incubator (Bioptechs Inc., Butler, PA.) and mounted on the stage of a Leica DMIRB inverted fluorescence microscope equipped with a Hamamatsu Color Chilled 3CCD camera (C5810 model) attached to a computer for data acquisition. The FCS2 incubator is a closed near-laminar flow, temperature-controlled perfusion chamber which facilitates direct observation and scanning of the cells on the coverslip during perfusion with solutions or cell suspensions. It was designed to maintain accurate thermal control and allow high-volume near-laminar flow perfusion. A fluid pathway was formed (Dimensions 0.5 mm × 14 mm × 25 mm) by separating the microaqueduct slide from the coverslip containing cells with a single silicone gasket with a rectangular bore, generating near-laminar flow conditions during perfusion. The stage incubator was initially connected to a peristaltic pump on the afferent side using 0.062 inch bore S/P medical grade silicone tubing (Fisher Scientific, Suwanee, GA), with a three way in-line tap closed to the incubator inlet. The efferent side was connected to waste. After connection of the electronic temperature control, the coverslips were perfused with RPMI to briefly buffer the cells at 37°C for fifteen minutes (flow rate of 0.5 ml/min). The inlet was then connected to the tube containing the appropriately treated monocyte suspension (3 mL). The efferent tubing was also connected to the same tube, and Di-I [30] labelled monocytes were recirculated across the tumor cells for 1 h, after which, the tubing was set up to the initial configuration and the cells were again washed with RPMI for 10 min to remove unbound monocytes. Individual still images in five randomly chosen fields of view were captured and stored on the computer. The number of monocytes (red) and tumor cells (green) were counted in each field of view and the mean monocyte/tumor cell ratio was calculated.

The wall shear stress was calculated using the momentum balance for a Newtonian fluid. The viscosity of water at 37°C was used as an approximation of the viscosity of the buffer used (RPMI 1640; 0.007 poise). As described in the following equation, the wall shear stress experienced by the monolayer is equal to: T = 3μQ/2a^2^b, where T=wall shear stress, μ= coefficient of viscosity (0.007 Poise), Q= volumetric flow rate (0.0083 cm^3^/s), a= half channel height (0.025 cm), and b=channel width (1.5 cm). The wall shear rate is given by T/μ. Experiments were conducted to maximize adherence in the model, within physiologically relevant shear rates (In post-capillary venules, shear rate has been reported to range between 35 and 560 s^-1 ^[31,32]. This range is believed to be characteristic of the stresses that a leukocyte must resist to form a stable adhesion with a vessel wall).

### Preparation of sFn blocking peptides

Based on the reported activities of the various fibrin(ogen) blocking peptides, four were selected which would possibly also inhibit fibrin mediated immunosuppression. Table 1 shows our laboratory designation, the amino acid sequence, its molecule of origin, and which molecule it binds to.

**Table 1:** Designation of sFn inhibitory peptides, sequences, molecule of origin and ligand

**Peptide #**	**Sequence**	**Molecule of Origin**	**Ligand**
P1	NNQKIVNLKEKVAQLEA	sFn (g-chain)	CD54 (1^st ^Ig)
P2	YKSMKKTTMKIIPFNRLTIF	sFn (g-chain)	α_M_β2 (αM I)
P3	KVILPRGGSVLVTC	CD54 (1^st^Ig)	sFn (y-chain)
P4	KFGDPLGYEDVIPEADREG	α_M_β2 (αM I)	sFn (g-chain)

#### Stock peptides

Lyophilized peptides (50% purity) were obtained from Sigma-Genoysys (The Woodlands, TX). Peptides 1,2, and 4 were each made up to a 100 μM stock concentration in PBS. Peptide 3 was first solubilized in a small amount of DMSO (on account of its hydrophobicity), followed by subsequent suspension in PBS, and was also made up to a 100 μM stock concentration. All peptides were divided into 50 μL aliquots, and stored at -20°C. Peptides were used at a working concentration of 4 μM.

### Effect of sFn blocking peptides on clot formation in purified fibrinogen and in recalcified citrated plasma

Experiments were performed to determine if the specific fibrin(ogen) adherence inhibitors also affected 1. fibrinogen clotting in the presence of thrombin, or 2. normal coagulation in re-calcified plasma.

1. Fibrinogen (0.5 mg/ml, 0.05 mmol/L), and thrombin (0.125 U/ml) were used at the concentration demonstrated to induce maximal inhibition of adherence and cytotoxicity in microplate assays. Thrombin (0.625 μl (stock 100 U/ml) was added to RPMI 1640 medium containing 25 μl (stock 10 mg/ml) thrombin alone or in the presence of 4 mmol/L specific peptide or peptide combination in a total volume of 1 ml. The tubes were left at room temperature and clotting was determined by observed gelation of the solution every 30 s, and the approximate clotting time was recorded. An additional control was also tested in which the fibrin polymerization inhibitor GPRP-NH2 was added to fibrinogen in the presence of P1-P4 prior to thrombin addition. The tube was left at room temperature to determine if clotting was observed.

2. The effect of the specific peptides on the clotting of re-calcified normal human plasma was also examined to determine if the normal coagulation cascade was inhibited in their presence. Plasma alone or in the presence of single or combined peptides P1-P4 (4 mM final concentration: 20 μl of 100 mM stock) was re-calcified with CaCl_2 _(50 μL; 25 mM stock) in a final volume of 500 μL. Tubes were left at room temperature and clotting time was then recorded.

### Peptide blocking of cellular adherence

After fluorescence labeling of each cell type, stock peptides were added to both confluent coverslips and/or 1 milliliter suspensions of monocytes (P1 and P2) and/or sFn solution (P3 and P4) for a final treatment concentration of 4 mM (40uL added to 1 mL suspension). Cells were treated with peptides for 20 minutes at room temperature, followed directly by washing of cells (by centrifugation and resuspension – monocytes, or perfusion with RPMI 1640 for 10 min – tumor cells). For sFn peptide treatment, peptides remained in the sFn solution during incubation. Monocyte adherence to tumor cells under flow conditions was then performed as described above.

### Peptide blocking of fluorescently labeled sFn adherence to A375 cells and monocytes

Oregon Green labeled fibrinogen was obtained from Molecular Probes (Oregon Green 488 human fibrinogen conjugate (F-7496)). Lyophilized stock fibrinogen (5 mg) was made up in RPMI 1640 to a total volume of 5 ml, aliquotted (25 μl) and stored at -20°C until time of experiment. For experiments, labeled sFn was prepared as described for sFn above, and incubated for 20 minutes at 37°C to allow sFn formation.

Stock peptides (P1 and P2) were added to both confluent coverslips containing unlabeled A375 cells and/or 1 milliliter suspensions of unlabeled monocytes and/or labeled sFn (P3 and P4) at a final concentration of 4 mM, for 20 minutes at room temperature, followed by washing in RPMI 1640. Peptides were retained in the sFn solution during adherence. Untreated or peptide pre-treated monocytes and/or tumor cells were incubated for 20 min with labeled-sFn (+/- peptide treatment as appropriate), and washed in RPMI 1640. The monocyte pellet was resuspended in 1 ml of phenol red free RPMI 1640 and 10 μl added to a microscope slide, coverslipped and sealed for microscopy. Coverslips containing tumor cells were inverted onto a microscope slide together with 50 μl of Pro-long Gold™ (Molecular Probes) mounting medium, and allowed to harden for microscopy. An Olympus BX61 was used (100× oil immersion objective) to observe samples and capture images. Using the same microscope settings to observe differences in fluorescence between samples, five representative fields were captured of each slide for each pre-treatment. Images were processed using Image Pro Plus™ (Media Cybernetics, Silver Spring, MD) and deconvolved using AutoDeblur™ deconvolution software (Media Cybernetics, Silver Spring, MD).

### Statistical analysis

**Figure 2:**
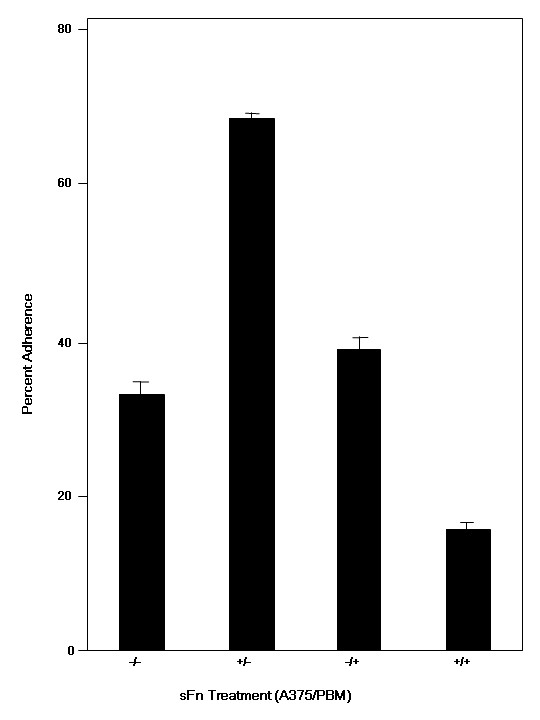
Effect of soluble fibrin on monocyte adherence to tumor cells. Calcein AM labeled PBM incubated with A375 cells after pre-treatment of A375 and/or PBM with RPMI or sFn prior to assay. sFn pre-treatment of tumor cells significantly increased adherence to untreated A375 cells compared to the untreated control (P < 0.01: n = 3). Preincubation of monocytes also marginally increased adherence to untreated A375 cells (P < 0.05: n = 3) compared to the untreated control, but to a significantly lower degree than with sFn treated A375 cells (P < 0.01 compared to monocyte sFn). sFn pre-treatment of both effector and target cells resulted in a significant inhibition of adherence (P < 0.05: n = 3) compared to the untreated control.

**Figure 3:**
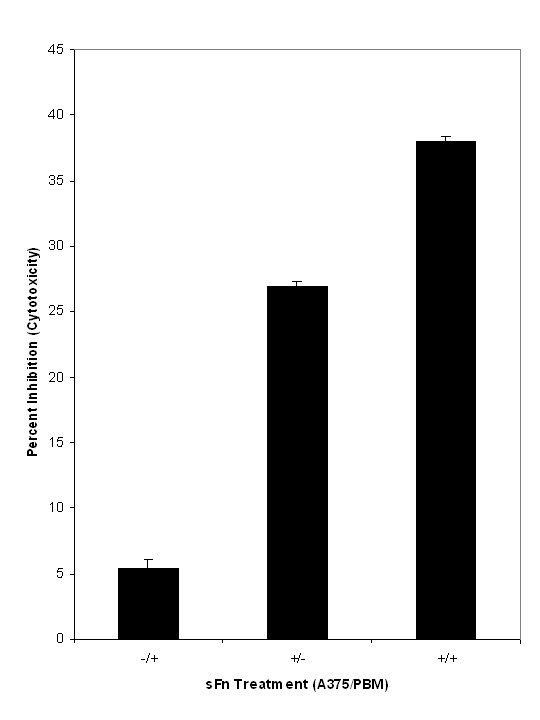
Effect of sFn pre-treatment on monocyte cytotoxicity against tumor cells. Calcein AM labeled PBM incubated with A375 cells after pre-treatment of A375 with or without sFn and pre-treatment of PBM with or without sFn prior to assay. sFn pre-treatment of monocytes was slightly inhibitory (P <0.05 compared to untreated control; n = 3). Significantly greater inhibition was observed when A375 cells were sFn pre-treated (P <0.01 compared to untreated and to monocyte treated cells; n = 3). Maximal inhibition of PBM cytotoxic activity occurred when both cell types were treated with sFn (P < 0.01 compared to untreated, monocyte treated or A375 treated cells: n = 3).

**Figure 6:**
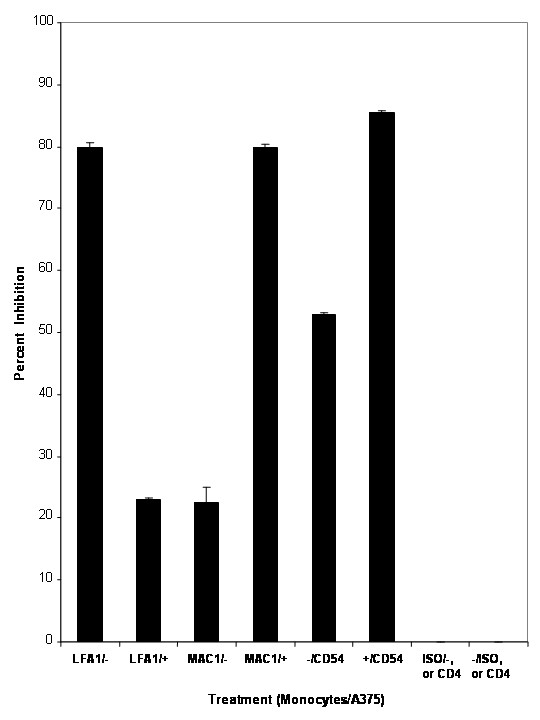
Effect of monoclonal anti-α_L_β2, -α_M_β2 and CD54 on monocyte adherence to tumor cells under flow conditions in the presence or absence of sFn. Anti-α_L_β2 inhibited monocyte adherence to untreated cells (P <0.05; n = 3), but was significantly (P < 0.01 compared to non-sFn treated cells; n = 3) less effective in blocking monocyte binding to sFn pre-treated tumor cells. Conversely, anti-α_M_β2 inhibited monocyte adherence to sFn pre-treated tumor cells to a significantly (P < 0.01) greater extent than to untreated tumor cells. Anti-CD54 inhibited monocyte adherence to untreated tumor cells by over 50%, and by over 80% when tumor cells were pre-incubated with sFn. Isotypic control IgGs or an irrelevant monoclonal antibody (CD4) did not affect monocyte adherence.

In static microplate adherence and cytotoxicity assays (Figures 2, 3 and 6) each data point was obtained as the mean +/- SD for three replicates. Each experiment was performed at least three times. Student's t-test for independent (unpaired) variables was used to determine significant differences between groups.

**Figure 7:**
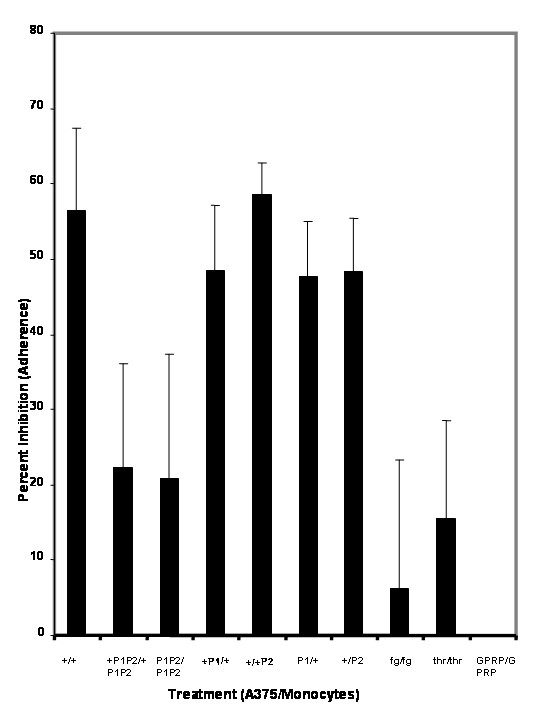
Effect of specific blocking peptides designated P1 (binds to CD54) and P2 (binds to α_M_β2) on sFn inhibition of monocyte/tumor cell adherence under flow conditions. (from left to right): sFn pre-treatment of monocytes and A375 cells (n = 25) significantly (P < 0.01 compared to untreated control; n = 30) inhibited monocyte adherence. Pretreatment of cells with P1 and P2 restored cell adherence to levels not significantly different to that of the untreated control (P < 0.05 to fibrin (n = 10); P > 0.05 to control). Pre-treatment of tumor cells with P1 and monocytes with sFn or tumor cells with sFn and monocytes with P2 inhibited adherence to a similar level to that of sFn treatment of both cells (P > 0.05 to fibrin; n = 5 in each case). Pretreatment of effector and tumor cells with fibrinogen (Fg), thrombin or GPRP did not significantly inhibit adherence (P > 0.05 compared to untreated cells; n = 5)

**Figure 8:**
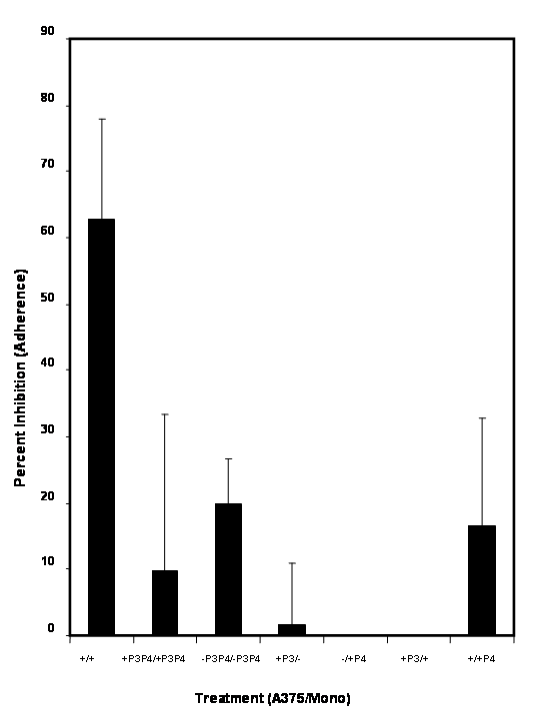
Effect of specific blocking peptides designated P3 (binds CD54 binding site on sFn) and P4 (binds to α_M_β2 binding site on sFn) on sFn inhibition of monocyte/tumor cell adherence under flow conditions. (from left to right): sFn treatment of monocytes and A375 cells (n = 25) significantly (P < 0.01 compared to untreated control; n = 10) inhibited cell adherence. Pretreatment of sFn with P3 and P4 restored cell adherence to levels not significantly different to the untreated control (P < 0.05 to fibrin; n = 5; P > 0.05 to control). Pre-treatment of tumor cells and/or monocytes with either P3+P4, P3 alone or P4 alone were not inhibitory (P > 0.05; n = 5 in each case, compared to untreated control).

For experiments investigating adherence under flow conditions (Figures 7 and 8), each experiment used monocytes from a single individual and the experiment was internally controlled on each occasion by inclusion of untreated and sFn/sFn treated cells. Each experiment was performed at least five times. Inhibition of adherence compared to the untreated control, as well as peptide blocking of sFn inhibition was determined by direct comparison with its own internal control in each experimental protocol. Thus, the number of untreated and sFn treated controls was greater than the number of tests for each single or combined peptide. Since there was also variation in monocyte adherence from individual donors, each peptide effect was compared to the whole population of either untreated or sFn treated control, using Student's t-test for independent (unpaired) variables.

## Results

### Effect of sFn on monocyte/tumor cell adherence in static microplate assays

Experiments were performed to determine the effect of sFn pre-incubation of either monocytes, tumor cells, or both on monocyte adherence to A375 melanoma cells in static microtiter plate assays (Figure 2). In the absence of sFn, monocytes adherence was 32.9 ± 1.3%. Pre-treatment of tumor cells with sFn significantly (P < 0.01 compared to untreated control) increased adherence (68.5 ± 0.7%). Addition of sFn treated monocytes to untreated tumor cells also increased adherence (38.75 ± 1%; P < 0.05 compared to untreated control), but to a significantly lower degree than tumor cell pretreatment with sFn (P < 0.01 compared to sFn treated tumor cells). However, when both monocytes and tumor cells were both pre-treated with sFn a pronounced inhibition of adherence was observed (15.95 + 1%; P < 0.01 compared to untreated control).

### Effect of sFn on monocyte cytotoxicity against tumor cells in static microplate assays

Monocyte cytotoxicity against A375 melanoma cells was measured in a static microplate assay (Figure 3). At an effector:target cell ratio of 20:1 monocyte cytotoxicity was 28.6 + 0.7%. Pre-treatment of A375 cells with sFn did not significantly (P >0.05) affect cytotoxicity (27.1 + 0.7%) compared to the untreated control, but a significant reduction (P <0.05) was observed when monocytes were sFn treated (20 + 0.7%). Maximal inhibition of cytotoxicity compared to untreated cells was observed when both effector and target cells were sFn treated (17.3 + 0.3%; P < 0.01 compared to Untreated control, sFn treated monocytes and to sFn treated A375 cells; n = 3).

### Effect of shear rate on monocyte adherence to tumor cells under flow conditions

**Figure 4:**
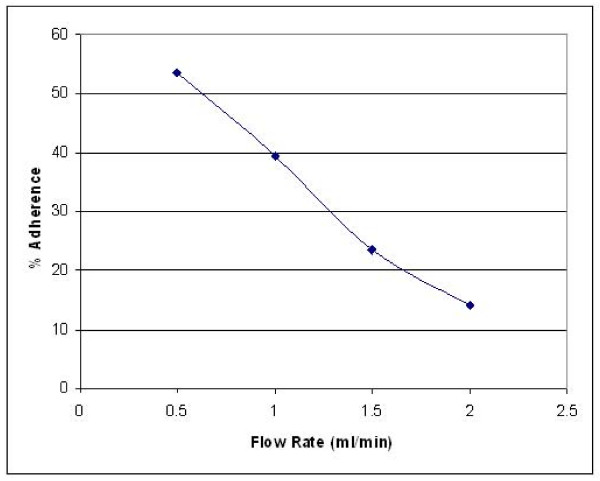
Effect of perfusion flow rate on monocyte adherence to tumor cells. Monocytes (1 × 10^6^/ml) were perfused across a monolayer of A375 cells attached to a coverslip in a perfusion stage incubator for 1 h at 37°C, and non-adherent cells were washed off by perfusion to waste for 10 min. Monocyte adherence was maximal at a flow rate of 0.5 ml/min, and linearly decreased as the flow rate was increased to 1, 1.5, and 2 ml/min.

**Figure 5:**
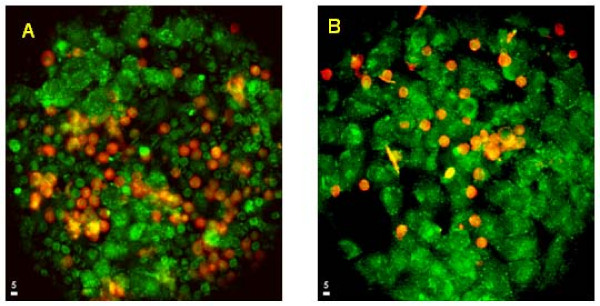
Tumor cells were grown on 40 mm coverslips to confluence and labeled with Calcein AM (green). Monocytes were labeled with DiI (C18; red), made to 1 × 10^6 ^cells/ml and continuously perfused across the tumor cells at 0.5 ml/min for 1 h. (Left) monocyte adherence to untreated A375 cells. (Right) monocyte (pre-treated with sFn) adherence to sFn pre-treated A375 cells. sFn pre-treatment of both effector and target cells inhibited monocyte adherence.

Initial experiments were conducted to maximize adherence in the model, within physiologically relevant shear rates. Figure 4 shows the percentage adherence (number of monocytes/field/number of tumor cells/field) at flow rates of 0.5 – 2.5 ml/min. Maximal monocyte adherence was observed at a flow rate of 0.5 ml/min corresponding to a shear stress of 0.93 dynes/cm^2^, with a corresponding shear rate of 132.9 s^-1^. This shear rate was within the range of the stresses (35 and 560 s^-1^) [31,32] which a leukocyte must resist to form a stable adhesion with a postcapillary vessel wall. Experiments investigating sFn inhibition of adherence were performed in further experiments at a flow rate of 0.5 ml/min. Figure 5 shows two representative images of monocytes (red) adherent to tumor cells (green). In Figure 5A untreated monocytes were adhered to untreated tumor cells. Less adherence was observed when both cells were pre-treated with sFn (B).

### Effect of sFn and monoclonal anti-α_L_β2, α_M_β2 and CD54 on monocyte/tumor cell adherence under flow conditions

Pre-treatment of monocytes with monoclonal anti-α_L_β2 antibody (5 μg/10^6^cells) inhibited their adherence to untreated A375 cells by 80 ± 7%. (Figure 6), whereas only 23 ± 0.3% inhibition was observed when A375 cells were also pre-treated with sFn (P < 0.05 compared to untreated A375 cells). Conversely, monocyte pre-treatment with anti-α_M_β2 monoclonal antibody only inhibited adherence by 22.5 ± 2.6% to untreated A375 cells, and by 80 ± 0.5% to sFn pre-treated tumor cells (P < 0.05 compared to untreated A375 cells). Incubation of A375 cells with anti-CD54 inhibited monocyte adherence of untreated monocytes by 53 ± 0.3%, and sFn pre-treated monocytes by 86 ± 0.2%, which were significantly greater (P < 0.05) compared to untreated monocytes). Pre-incubation of either cell type with appropriate isotypic control or an irrelevant antibody (CD4) did not significantly (P > 0.05) inhibit adherence.

### Effect of α_M_β2 and CD54 specific blocking peptides on thrombin induced fibrin polymerization

Experiments were performed to determine if fibrin(ogen) blocking peptides affected the ability of fibrinogen to clot. Using purified fibrinogen in the absence or presence of P1 + P2, P3 + P4, or all four peptides together, clotting was observed within approximately 5 minutes. Addition of GPRP-NH2 to purified fibrinogen prior to thrombin addition failed to clot within 15 minutes, indicating the production of soluble, rather than polymerized fibrin. Using no treatment, or the same combinations of peptides, clotting of recalcified plasma was observed under all conditions in approximately 4 minutes.

### Effect of sFn, α_M_β2 and CD54 specific blocking peptides on sFn inhibition of monocyte/tumor cell adherence under flow conditions

Figure 7 shows the effect of two specific peptides, designated P1 and P2 on sFn inhibition of monocyte adherence to A375 melanoma cells in a flowing microscope stage incubator. P1 represents the sFn major binding site for CD54 and P2 represents the sFn major binding site for α_M_β2. From the left, the first bar (+/+) shows the mean (+SD) inhibition when both monocytes and tumor cells were pre-incubated for 20 min with sFn. SFn considerably reduced monocyte adherence (P < 0.05 compared to untreated cells). When both monocytes (which express α_M_β2 and some CD54) and tumor cells were pre-treated with both P1 and P2 followed by sFn prior to assay (Bar2 +P1P2/+P1/P2) sFn inhibition was considerably reduced to a level which was not significantly different to the untreated control, as was also observed when cells were treated with peptides in the absence of sFn (Bar3; P1P2/P1/P2). These results suggest that by blocking the major receptor sites for sFn on α Mβ2 and CD54 binding of sFn is blocked, allowing α Lβ2 (LFA1 which does not bind to sFn) and α_M_β2 to bind to CD54 restoring adherence. If A375 cells are treated with P1 to block CD54 binding to fibrin (even when sFn was added subsequently) and monocytes are treated with sFn, inhibition was still observed (Bar4; +P1/+) since CD54 was blocked by P1. Similarly, inhibition was still observed when monocytes were treated with P2 to block sFn binding to α Mβ2, and tumor cells were treated with sFn (Bar 5; +/+P2). As controls, tumor cells were incubated with P1 (but not sFn) and monocytes were treated with sFn. Inhibition was still observed because P1 blocked sFn coated α Mβ2 binding (Bar 6; P1/+). Similarly, inhibition was observed when P2 blocked sFn binding to monocytes and tumor cells were treated with sFn (Bar 7; +/P2). The final 3 bars (Fg/Fg, thr/thr, GPRP-NH2/GPRP-NH2) showed that no significant (P > 0.05 compared to untreated cell adherence) inhibition was observed when cells were treated with soluble fibrinogen, thrombin, or GPRP-NH2). These results demonstrate the peptides P1 and P2 are effective in inhibiting sFn inhibition of monocyte adherence by a mechanism involving its blocking of CD54 to α_M_β2.

Figure 8 shows the effect of two specific peptides, designated P3 and P4 on sFn inhibition of monocyte adherence to A375 melanoma cells in a flowing microscope stage incubator. P3 represents the CD54 major binding site for sFn and P4 represents the α Mβ2 major binding site for sFn. From the left, the first bar (+/+) shows the mean (+SD) when both monocytes and tumor cells were pre-incubated for 20 min with sFn. SFn considerably reduced monocyte adherence (P < 0.05 compared to untreated cells). Pre-treatment with sFn with P3 and P4 prior to its incubation with tumor cells and monocytes (which should block its binding) resulted in a marked increase in cell adherence, which was not significantly different than the untreated control (Bar 2; +P3P4/+P3P4;P < 0.05), thus reversing sFn inhibition of adherence. As expected, addition of peptides P3 and P4 to cells did not significantly affect adherence (Bar 3; -P3P4/-P3P4; P > 0.05 compared to untreated). When sFn was treated with P3 and incubated with tumor cells, and monocytes were untreated, little or no inhibition was observed, because α Mβ2 could still bind to CD54 directly (Bar4; +P3/-). Similarly, when sFn was pre-treated with P4 and incubated with monocytes, and tumor cells were untreated, no inhibition was observed, because tumor cells would not bind P3-sFn, and the sFn on the monocytes could bind to CD54 on the tumor cells (Bar 6; +P3/+). Similarly, when sFn was pre-treated with P4 and incubated with monocytes, and tumor cells were incubated with sFn, no inhibition was observed, because the free α Mβ2 could still bind to sFn on the tumor cells (Bar 7; +/+P4).

These results show that blocking of sFn with peptides representing its major CD54 and α Mβ2 receptor sites effectively inhibited its ability to block adherence of monocytes to tumor cells.

### Effect of α_M_β2 and CD54 specific blocking peptides on sFn adherence to monocytes and tumor cells

**Figure 9:**
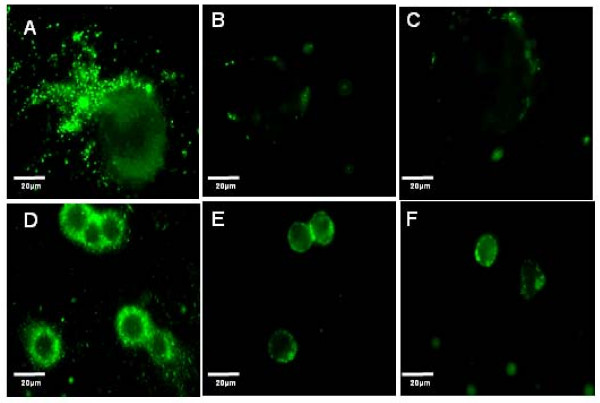
Oregon Green labeled fibrinogen (0.5 mg/ml; Molecular Probes, Eugene, OR) was treated with thrombin (1.25 U) in the presence of 4 mM GPRP-NH2 to produce fluorescently labeled sFn. A375 cells were incubated with labeled sFn for 20 min in a Bioptechs FCS2 enclosed stage incubator. The residual sFn was washed away by perfusion and the cells were imaged on an Olympus BX61 fluorescence microscope equipped with a long pass 535 nm dichroic filter. Considerable binding of sFn was observed. A is a representative image showing tumor cell sFn binding. In contrast, little or no binding was observed when cells were pre-incubated with peptides P1 + P2 (B), or sFn with P3 + P4 (C). Similarly, sFn bound readily to monocytes (D), but was inhibited when cells were pre-incubated with P1 + P2 (E), or sFn was pre-treated with P3 + P4 (F).

Having demonstrated that specific sFn blocking peptides reversed sFn inhibition of monocyte/tumor cells adherence, experiments using fluorescently labeled fibrinogen to prepare sFn were performed to observe whether sFn binding to cells was also decreased. After sFn binding (+/-) peptide pre-treatment, slides were observed on an Olympus BX61 fluorescence microscope equipped with an Olympus (COOL-1300QS) digital camera for image acquisition. Oregon Green fluorescence was detected using a 535 nm long-pass dichroic filter. After setting up the microscope to detect tumor cells or monocytes, all settings were kept identical for subsequent slides. Figure 9 shows binding of FITC-sFn to A375 cells (A). Fluorescent sFn bound strongly to both the cell types. However, when tumor cells were pre-treated with P1 + P3 (B), or sFn was pre-treated with P3 + P4 (C), almost no binding was observed. Similarly, sFn bound strongly to monocytes (D). Monocyte Pre-incubation with P1 + P2 considerably reduced sFn fluorescence, as did sFn pre-incubation with P3 + P4, demonstrating that the blocking peptides cause reduced sFn binding to cells.

## Discussion

Our results clearly demonstrate that sFn inhibits both monocyte adherence and cytotoxicity against tumor cells. Our previous work [33] demonstrated that sFn coated tumor cells enhanced metastasis in an experimental metastasis model. Further studies using monoclonal blocking antibodies showed that sFn binds to the β2 integrin receptor, CD54, which is present on many cancer cells. Other reports have demonstrated that fibrinogen binds to α_M_β2 on leukocytes [22,34].

In microplate monocyte/tumor cell adherence assays, monocytes adhered to tumor cells (Figure 1). Pre-treatment of tumor cells with sFn considerably enhanced monocyte binding. This is consistent with the report by Ugarova *et al *[22], showing increased monocyte binding to fibrinogen bound endothelial cells. Indeed, this has been postulated as a mechanism of increased immune cell homing to inflammatory sites. A small, but non-significant increase in monocyte adherence was also observed when monocytes were pre-incubated with sFn. However, monocyte adherence was inhibited by over fifty percent when both cell types were sFn treated prior to assay. Since sFn levels are elevated in many cancer patients, peripheral blood monocytes and circulating tumor cells undergoing metastasis would likely be coated in sFn, reducing their ability to adhere to each other.

Since adherence is a necessary step in most cytotoxic effector pathways, cellular cytotoxicity assays were performed to determine if monocyte killing of tumor cells was similarly inhibited by sFn When both cell types were untreated, specific monocyte cytotoxicity was 28.6 ± 0.7 (Figure 3). A small, but non significant (P >0.05) inhibition was observed when monocytes were sFn pre-treated. However, a greater inhibition (P < 0.05 compared to untreated cells) was observed when untreated monocytes were incubated with sFn treated tumor cells. This is interesting, since increased monocyte adherence was observed under similar conditions (Figure 1). This suggests that although sFn bound to tumor cells increases monocyte adherence, it may inhibit subsequent signaling to allow delivery of the cytotoxic lethal hit to the tumor cells. Furthermore, these results are consistent with our previously published data showing increased metastasis when sFn treated tumor cells were injected into warfarinized mice [33]. SFn pre-treatment of both monocytes and tumor cells resulted in the greatest inhibition of cytotoxicity (P < 0.05 compared to both untreated and sFn treated tumor cells), which is consistent with the observed decrease in adherence under similar conditions. Taken together, these results would suggest that, *in vivo*, elevated levels of circulating sFn would result in its binding to both tumor cells and monocytes, leading to an ongoing immunosuppression.

To investigate the mechanism of sFn inhibition, monoclonal blocking antibodies directed against monocyte α_L_β2 and α_M_β2, or tumor cell CD54 were incubated with untreated or sFn treated monocytes and tumor cells (Figure 2). Pre-incubation of monocytes with blocking anti-α_L_β2 inhibited adherence to tumor cells by over eighty percent. However, inhibition by anti-α_L_β2 was significantly (P < 0.01) reduced when tumor cells were sFn treated. Conversely, monocyte pre-treatment with blocking anti-α_M_β2 had relatively little effect on adherence to untreated tumor cells, but significant blocking was observed if the tumor cells were sFn treated. Although this antibody has not been reported to block fibrin(ogen) binding to α_M_β2, it is likely since the epitope for this clone is on the α_M _I-domain, which is proximal to the fibrin(ogen) γ-chain binding site on α_M_.

These data suggest that, in the absence of sFn, unstimulated monocyte adherence is mediated predominantly via α_L_β 1, whereas α_M_β2 seems to predominate in binding to sFn treated tumor cells. This is consistent with previous observations that fibrin(ogen) binds to α_M_β2 [34]. These results were further supported by the observation that pre-treatment of tumor cells with blocking anti-CD54 inhibited untreated monocyte adherence and was even more effective in blocking adherence of sFn treated monocytes. This observation is supported by the observations of Gardiner and D'Souza [35]. This clone of anti-CD54 (84H10) inhibits both β2 integrin and fibrinogen binding to CD54. Isotypic murine immunoglobulin isotype or an irrelevant monoclonal antibody (anti-CD4) had no effect on monocyte – tumor cell adherence in the presence or absence of sFn.

To further investigate the mechanism of sFn inhibition of monocyte/tumor cells adherence, four peptides were chosen which had previously been reported to represent major fibrin(ogen) binding sites on CD54 (P1) and α_M_β2 (P2) for fibrin(ogen), and the fibrin(ogen) γ-chain binding sites for CD54 (P3) or α_M_β2 (P4). Blocking of the fibrin(ogen) binding sites on both monocytes (α_M_β2) and tumor cells (CD54), prior to sFn treatment significantly (P < 0.05) reduced sFn mediated inhibition of monocyte/tumor cell adherence under flow conditions (Figure 7). Blocking of both the CD54 and α_M_β2 binding sites on sFn again significantly (p < 0.05) reduced the ability of sFn to inhibit monocyte adherence (Figure 8). In further experiments Oregon Green-labeled sFn was incubated with tumor cells or monocytes alone or after appropriate cell or sFn blocking peptide treatment. Fluorescence microscopy (using exactly the same microscope settings for all slides) showed a considerable reduction in sFn binding to either cell type after appropriate cell or sFn treatment with either P1 + P2, or P3 + P4, confirming that peptide treatment inhibits sFn binding to the cells (Figure 9). Further evidence of the specificity of the blocking peptides was provided in experiments demonstrating their inability to inhibit normal fibrinogen clotting in a pure system and in recalcified plasma.

**Figure 10:**
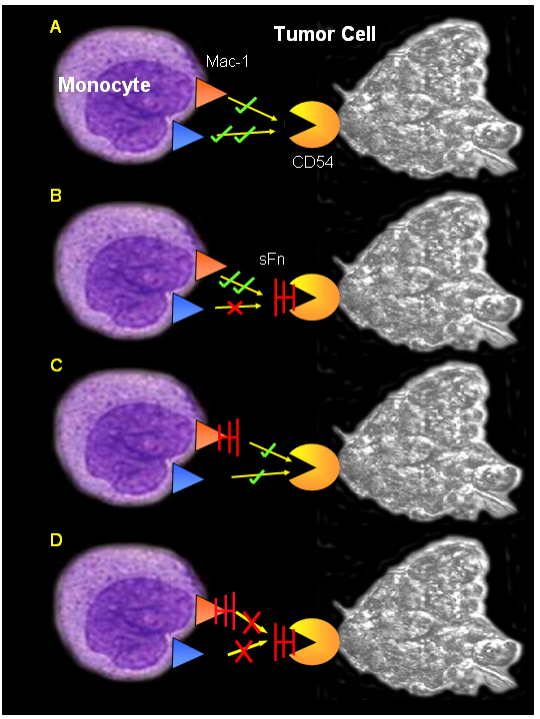
Schematic diagram summarizing the proposed mechanism of sFn mediated inhibition of monocyte adherence (and consequently cytotoxicity) to tumor cells. A. α_L_β2 (orange) binds preferentially compared with α_M_β2 (blue) to tumor cell CD54. B. Pre-treatment of tumor cells with sFn inhibits α_L_β2 (α_L_β2 does not bind fibrin(ogen) binding, but enhances α_M_β2 mediated adherence). C. Pre-treatment of monocytes with sFn (sFn binds to α_M_β2) allows adherence by both α_L_β2 and sFn bound α_M_β2 to tumor cell CD54. D. Pre-incubation of monocytes and tumor cells with sFn inhibits both α_L_β2 and α_M_β2 binding to sFn coated CD54.

Figure 10 is a schematic diagram summarizing the results of this study. The presence of sFn on tumor cells enhances monocyte binding, probably by upregulating α_M_β2 binding to sFn coated CD54 (B), which is consistent with our results and with previous reports [21,22]. Pre-treatment of monocytes (but not tumor cells) with sFn does not affect monocyte adherence, probably because free tumor cell CD54 is still available to bind monocyte α_L_β2 (C). Pretreatment of both cells with sFn inhibits both α_L_β2 and α_M_β2 mediated adherence (D), which is the most likely situation *in vivo*, in patients with elevated levels of circulating sFn.

Extrapolation of these results to the physiological setting would suggest that, in cancer patients with elevated levels of sFn, monocytes and circulating tumor cells would bind sFn, resulting in the inability of monocytes to adhere to, and kill, tumor cells, resulting in enhanced metastasis. The use of fibrin blocking peptides may represent a novel class of therapeutic agents to reduce sFn mediated immunosuppression, and decrease metastasis in many cancers, while avoiding the negative bleeding problems commonly associated with anticoagulant therapies.

## Authors' contributions

JB: Designed the project, performed data and statistical analysis, and wrote the manuscript.

BW: Performed peptide-flow adherence assays, data collection, and assisted in manuscript preparation.

JV: Performed monocyte adherence-tumor cell assays.

JD: Performed antibody-blocking of monocyte adherence to tumor cells under flow conditions.

SP: Performed static microplate monocyte adherence assays.

PP: Performed static microplate cytotoxicity assays.

All authors have read and approved the final manuscript.
